# ENPP2 promotes progression and lipid accumulation via AMPK/SREBP1/FAS pathway in chronic lymphocytic leukemia

**DOI:** 10.1186/s11658-024-00675-6

**Published:** 2024-12-27

**Authors:** Liyan Lu, Xinting Hu, Yang Han, Hua Wang, Zheng Tian, Ya Zhang, Xin Wang

**Affiliations:** 1 Department of Hematology, Shandong Provincial Hospital Affiliated to Shandong First Medical University, Add: No.324, Jingwu Road, Jinan, 250021 Shandong China; 2 Department of Hematology, Shandong Provincial Hospital, Shandong University, Jinan, 250021 Shandong China; 3 Taishan Scholars Program of Shandong Province, Jinan, 250021 Shandong China

**Keywords:** Chronic lymphocytic leukemia, Lipidomics, Lipid metabolism, ENPP2, PF-8380, AMPK, LPL

## Abstract

**Background:**

Disorders of lipid metabolism are critical factors in the progression of chronic lymphocytic leukemia (CLL). However, the characteristics of lipid metabolism and related regulatory mechanisms of CLL remain unclear.

**Methods:**

Hence, we identified altered metabolites and aberrant lipid metabolism pathways in patients with CLL by ultra-high-performance liquid chromatography-mass spectrometry-based non-targeted lipidomics. A combination of transcriptomics and lipidomics was used to mine relevant target molecule and downstream signaling pathway. In vitro cellular assays, quantitative real-time polymerase chain reaction (qRT-PCR), western blot, fluorescent staining, RNA sequencing, and coimmunoprecipitation were used to monitor the molecular levels as well as to explore the underlying mechanisms.

**Results:**

Significant differences in the content of 52 lipid species were identified in CLL samples and healthy controls. Functional analysis revealed that alterations in glycerolipid metabolism, glycerophospholipid metabolism, sphingolipid metabolism, and metabolic pathways had the greatest impact on CLL. On the basis of the area under the curve value, a combination of three metabolites (phosphatidylcholine O-24:2_18:2, phosphatidylcholine O-35:3, and lysophosphatidylcholine 34:3) potentially served as a biomarker for the diagnosis of CLL. Furthermore, utilizing integrated lipidomic, transcriptomic, and molecular studies, we reveal that ectonucleotide pyrophosphatase/phosphodiesterase 2 (ENPP2) plays a crucial role in regulating oncogenic lipogenesis. ENPP2 expression was significantly elevated in patients with CLL compared with normal cells and was validated in an independent cohort. Moreover, ENPP2 knockdown and targeted inhibitor PF-8380 treatment exerted an antitumor effect by regulating cell viability, proliferation, apoptosis, cell cycle, and enhanced the drug sensitivity to ibrutinib. Mechanistically, ENPP2 inhibited AMP-activated protein kinase (AMPK) phosphorylation and promoted lipogenesis through the sterol regulatory element-binding transcription factor 1 (SREBP-1)/fatty acid synthase (FAS) signaling pathway to promote lipogenesis.

**Conclusions:**

Taken together, our findings unravel the lipid metabolism characteristics of CLL. Moreover, we demonstrate a previously unidentified role and mechanism of ENPP2 in regulation of lipid metabolism, providing a novel therapeutic target for CLL treatment.

**Supplementary Information:**

The online version contains supplementary material available at 10.1186/s11658-024-00675-6.

## Introduction

Chronic lymphocytic leukemia (CLL), a malignant B-cell tumor, is the most common form of adult leukemia in western countries [1, 2]. As part of plastic and context-dependent metabolic reprogramming triggered by both oncogenic and environmental stimuli, cancer cells and other cell types use a variety of strategies to access lipids in the tumor microenvironment [3, 4]. It has been observed that CLL cells could rapidly take up fatty acids to promote their proliferation [5]. A close association between altered lipid metabolism and pathogenicity is supported. In this context, particular lipid profiles are evolving as distinct biomarkers with diagnostic capabilities. On the other hand, with the development of targeted therapeutic agents, there have been significant improvements in CLL treatment [6–8], but CLL currently remains as a challenging hematologic neoplasm. Discovering innovative therapeutic targets for CLL remain significant imperatives that require attention.

Ectonucleotide pyrophosphatase/phosphodiesterase 2 (ENPP2), an adipocyte-derived lysophospholipase D, played an extensive role in many metabolisms [9–11]. ENPP2 expression is upregulated in corpulence patients and mice and is associated with insulin resistance and impaired glucose tolerance [11, 12]. ENPP2 has been described to be engaged in several solid neoplasms, such as chondrosarcoma [13], breast cancer [14], hepatocellular carcinoma [15], and pancreatic cancer [16], and has been mentioned in multiple myeloma [17]. Nevertheless, the effects of ENPP2 inhibition in CLL remain poorly understood.

Herein, we integrated lipidomics and transcriptomics to investigate the lipid metabolic features of CLL. In addition, our study was the first investigation on the role of ENPP2 in the tumorigenesis of CLL. The biological processes involved were examined through loss-of-function and gain-of-function assays, and unraveling the regulatory mechanism in CLL. In conclusion, our results will inform a CLL treatment strategy.

## Materials and methods

### Metabolomics data processing

Data were analyzed as described previously [18, 19]. Details of the methods is provided in the Supplementary Material. Fold change (FC) > 2.0 or < 0.5, q value < 0.05 and variable importance in projection (VIP) > 1 was taken as the screening conditions to obtain significantly different metabolites. By using LipidSearch 4.2, all differential feature ions were annotated. Using the R package (heatmap), a heatmap was created using the annotated differential lipids. On the basis of the Kyoto encyclopedia of genes and genomes (KEGG) database, a pathway enrichment study was done on LIPEA, and 25 enhanced pathways were displayed in a scatter plot. The area under the curve (AUC) was calculated and illustrated using GraphPad Prism 9.0.

### Transcriptome data analysis

RNA extraction was performed on cell samples using RNAiso Plus from TaKaRa (Dalian, China). Subsequently, Huada Gene Technology Co. Ltd (Shenzhen, China) analyzed cell samples using the Illumina HiSeq 4000 platform. KEGG pathway analysis was performed on the screened differentially expressed genes (DEGs) to acquire the biological functions of these DEGs.

### Cell lines and reagents

The MEC-1 cell line, a human p53 deleted/mutated CLL cell line, was obtained from the Moores Cancer Center at the University of California, San Diego. The human CLL cell line, EHEB, was derived from American Type Culture Collection (ATCC, Manassas, VA, USA). These cells were cultured in supplemented IMDM, RPMI-1640 medium with 10% heat-inactivated fetal bovine serum (FBS) obtained from Gibco, MD, USA, alongside 1% penicillin/streptomycin mixture, 2 mM L-glutamine, and incubated under ideal conditions of 37 °C with 5% CO_2_. Regular screening for mycoplasma infection was conducted on all cells. ENPP2 inhibitor PF-8380 (S8218, Selleck, Shanghai, China) and Ibrutinib (PCI-32765, MCE, Shanghai, China) were soluble in dimethyl sulfoxide (DMSO; Solarbio, Beijing, China).

### Patient specimens

The medical ethics committee of Shandong Provincial Hospital approved this study and informed consent was acquired from each patient. The participants in this study were 82 patients diagnosed and treated in the department of hematology at Shandong Provincial Hospital, and their blood samples were collected. The criteria for diagnosing CLL were based on the revised International Workshop on Chronic Lymphocytic Leukemia (IWCLL) [20]. Patients’ peripheral blood mononuclear cells (PBMCs) were extracted using the FicollHypaque density gradient method according to previously reported methods [21, 22].

### RNA isolation and quantitative real-time PCR

The purification of total RNA was carried out using RNAiso Plus (TaKaRa, Dalian, China). Reverse transcription was carried out utilizing a reverse transcription kit from the same source. In adherence to the manufacturer’s instructions, quantitative real-time polymerase chain reaction (qRT-PCR) was conducted, and the results were analyzed through Light cycler 480 software. Primer sequences were as follows: ENPP2-F: ACTTGTGATGATAAGGTAGAGCCA; ENPP2-R: CTGTAGACCCTTTTGTATGAAGCC; LPL-F: AGTAGCAGAGTCCGTGGCTA; LPL-R: ATTCCTGTTACCGTCCAGCC; GAPDH-F: 5′-GCACCGTCAAGGCTGAGAAC-3′; GAPDH-R: 5′-TGGTGAAGACGCCAGTGGA-3′. Details of the methods is provided in the Supplementary Materials.

### Plasmid mediated regulation of ENPP2

The sequences for ENPP2 shRNAS were as follows: shENPP2#1, 5′-GCAGCAAAGTCATGCCTAATA-3′; shENPP2#2, 5′-GCAGTGCTTTATCGGACTAGA-3′. The knockdown plasmids were synthesized by GenePharma (Shanghai, China). GenePharma (Shanghai, China) synthesized and purified corresponding negative control plasmids. The sequence of ENPP2 lvRNA was 5′-CGCAAATGGGCGGTAGGCGTG-3′. The pENTER-ENPP2-Flag/His plasmid was purchased from ViGene Biosciences Inc (Shandong, China). Lipofectamine 3000 reagent (Invitrogen) was used to transiently transfect plasmids into cells.

### Cell proliferation assays

The procedure was performed as described previously [21, 23]. Cell Counting Kit-8 (CCK-8) (Dojindo, Kumamoto, Japan) was used. Details of the methods is provided in the Supplementary Materials.

### Analysis of cell apoptosis and cell cycle

The procedure was performed as described previously [21, 23]. The reagents used were as follows: Annexin V-PE/7AAD Kit (BD Biosciences, Bedford, MA, USA); PI/RNase Staining Buffer (BD Biosciences, Bedford, MA, USA). Details of the methods is provided in the Supplementary Materials.

### Elisa assay

Collect the cell supernatant after treating the cells separately and the concentration of lysophosphatidic acid (LPA) was measured using human LPA ELISA Kit (LANSO, China).

### Western blotting

The western blot procedure was performed as described previously [21, 23]. The primary antibodies used were as follows: ENPP2, LPL (Santa Cruz Biotechnology, USA), c-myc, Cyclin D1, CDK4, p21, p27, Bcl-2, Bax (Cell Signaling Technology, USA), AMPK, p-AMPK, SREBP1, FAS (abcam, USA), α-tubulin, and GAPDH (Zhongshan Goldenbridge, Beijing, China). Secondary antibodies were obtained from from Zhongshan Goldenbridge, Beijing, China. Details of the methods is provided in the Supplementary Materials.

### Triglyceride (TG) assay

The quantification of TG content in CLL cells was performed in accordance with the instructions provided by the manufacturer. A triglyceride quantification kit (BC0625, Solarbio, China) was utilized for this purpose.

### Lipid staining assay

Cells were incubated with BODIPY 493/503 (HY-W090090, MCE, USA) at 37 °C for 30 min in the incubator, with 4′,6-diamidino-2-phenylindole (DAPI; Beyotime, Shanghai, China) for 5 min at room temperature and observed under a fluorescent microscope.

### Co-immunoprecipitation (Co-IP) assay

Lysis of cells with Co-IP lysis solution. The resulting lysate was subjected to centrifugation, and the supernatant was treated with 1–3 ug of primary antibody before being shaken and incubated at 4 °C overnight. Subsequently, Protein A/G agarose (Santa Cruz Biotechnology, USA) were added to the antibody-treated buffer and incubated for 1 h at 4 °C to facilitate antibody binding. Phosphate buffered saline (PBS) was used to wash the beads three times before heating at 100 °C to denature the proteins. Detection of target proteins was carried out using western blotting.

### Statistical analysis

The data in this paper underwent statistical analysis using SPSS 26.0 software and GraphPad Prism 9.0 statistical software. The study presents the mean ± standard deviation (SD) of results obtained from three distinct experiments. Student’s *t*-test and Mann–Whitney *U* test were used for direct comparisons, while multigroup comparisons were carried out using one-way analysis of variance (ANOVA) or two-way ANOVA. The significance threshold was established at * p < 0.05 to declare statistical significance.

## Results

### Untargeted metabolomics demonstrate significant differences in lipid metabolites between patients with CLL and healthy controls

**Fig. 1:**
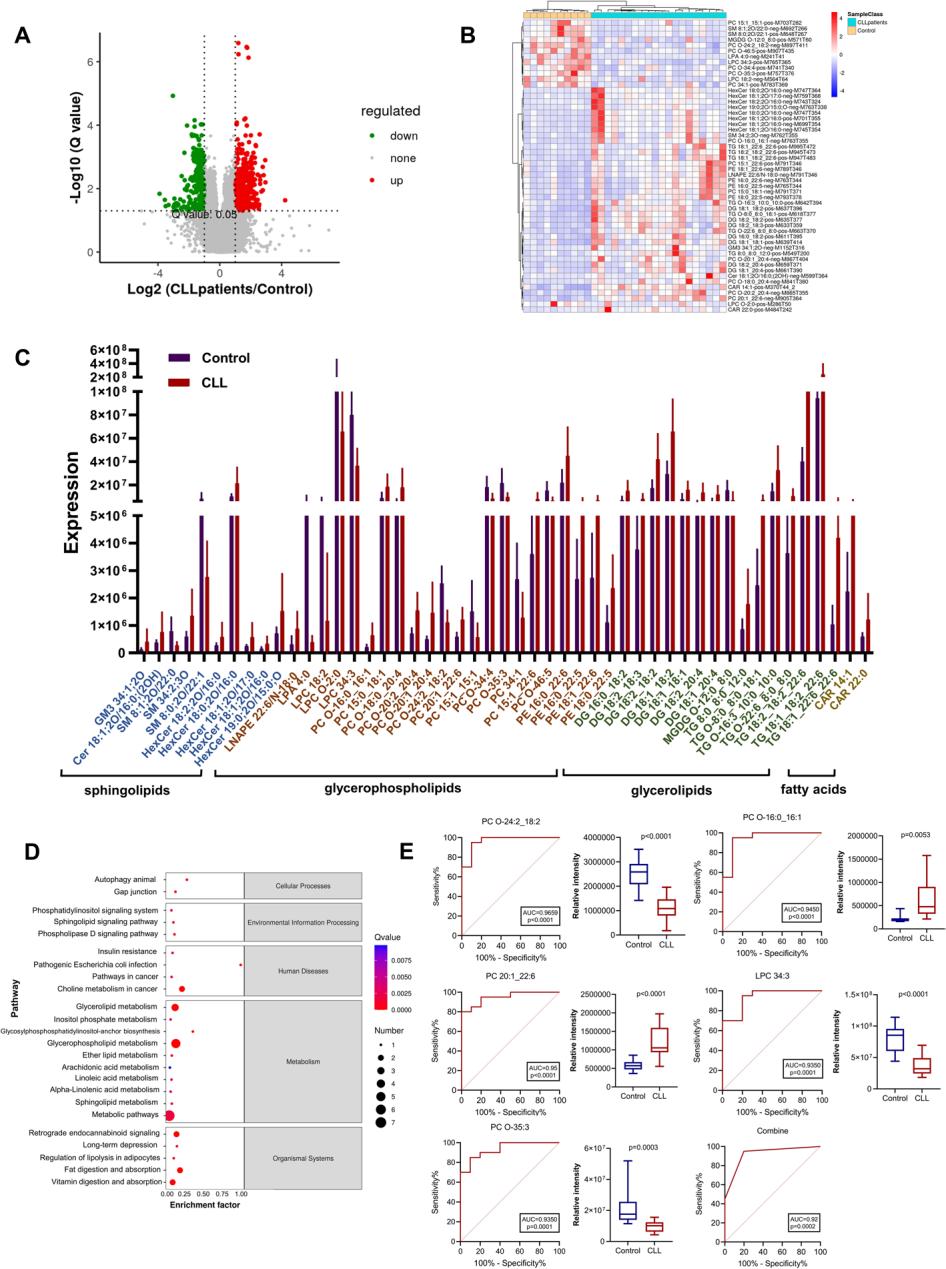
GC/LC–MS based multivariate data analysis of serum data between CLL groups and healthy controls. **A** Differential metabolite volcano map. **B** Heat map clustering of serum metabolites from the case and healthy control groups according to liquid chromatography-mass spectrometry (LC–MS). Significantly upregulated metabolites are shown in red (FC ≥ 1, *p* < 0.05), significantly downregulated metabolites are shown in blue (FC ≤ −1, *p* < 0.05), and non-significantly different metabolites are shown in gray. **C** Identification and annotation of metabolites. **D** Enrichment analysis of metabolites was performed. The scatter plot shows the most variable metabolic pathways. **E** receiver operating characteristic (ROC) curves of metabolic products

To investigate the differences of lipid metabolites in patients with CLL and normal subjects, we retained patients with CLL blood supernatants for untargeted lipidomics profiling. Patient information and commonly associated indicators of clinical lipid metabolism are shown in Supplementary Table 1. In our study, we employed univariate analysis techniques, specifically assessing fold-change and utilizing *t*-test statistical testing with BH correction to derive q-values. Further, we integrated the VIP metric generated from multivariate statistical analysis, PLS-DA (Fig. S1A-C). The volcano plot (Fig. 1A) revealed a total of 913 differential feature ions, displaying apparent patterns of both upregulation and downregulation. Remarkable variations were observed in the levels of sphingolipids (SP), glycerolipids (GL), glycerophospholipids (GP), and fatty acids (FA) in patients afflicted with CLL, in comparison with their healthy counterparts. To illustrate the expression of the 52 annotated differential metabolites between CLL and healthy control groups, a clustering heatmap was utilized, as depicted in Fig. 1B. To pinpoint the pathways with strong differential metabolite enrichment, we conducted a thorough analysis of the annotated results using enrichment analysis techniques. A comprehensive analysis has revealed the annotation of 52 differential metabolites, with 40 indicating an upregulation and 12 indicating a downregulation, as evidenced by Fig. 1C. Metabolic analysis software MetaboAnalyst v5.0 was used to analyze 52 different metabolite pathways between patients with CLL and control samples, indicating significant enrichment of 24 pathways. The KEGG enrichment scatter plot **(**Fig. 1D**)** shows that the metabolites differing between CLL patients and healthy controls were mainly labeled as enriched in glycerolipid metabolism, inositol phosphate metabolism, glycerophospholipid metabolism, ether lipid metabolism, sphingolipid metabolism, and metabolic pathways.

Then, we conducted the comprehensive evaluation of the lipids to explore potential lipid biomarkers for diagnosing CLL. Through this investigation, five metabolites were discovered to have notable diagnostic significance, as evidenced by the top AUC values (Fig. 1E). Notably, all of the top five lipids exhibited AUC values above 0.93. Especially, PC O-24:2_18:2, which had the highest AUC value of 0.965 [95% confidence interval (CI) 0.9006–1.000]. Three of these metabolites were chosen as combinational potential biomarkers for CLL. The model equation established after removing the confounding factor was Y = −2.575 + 2.126*PC O-24:2_18:2 + 3.544*LPC 34:3 + 3.174* PC O-35:3. The area under the curve (AUC) value of these biomarkers was 0.92 (95% CI 0.764–0.997), which was diagnostically significant.

### Integrative analysis of metabolomics and transcriptomics

**Fig. 2:**
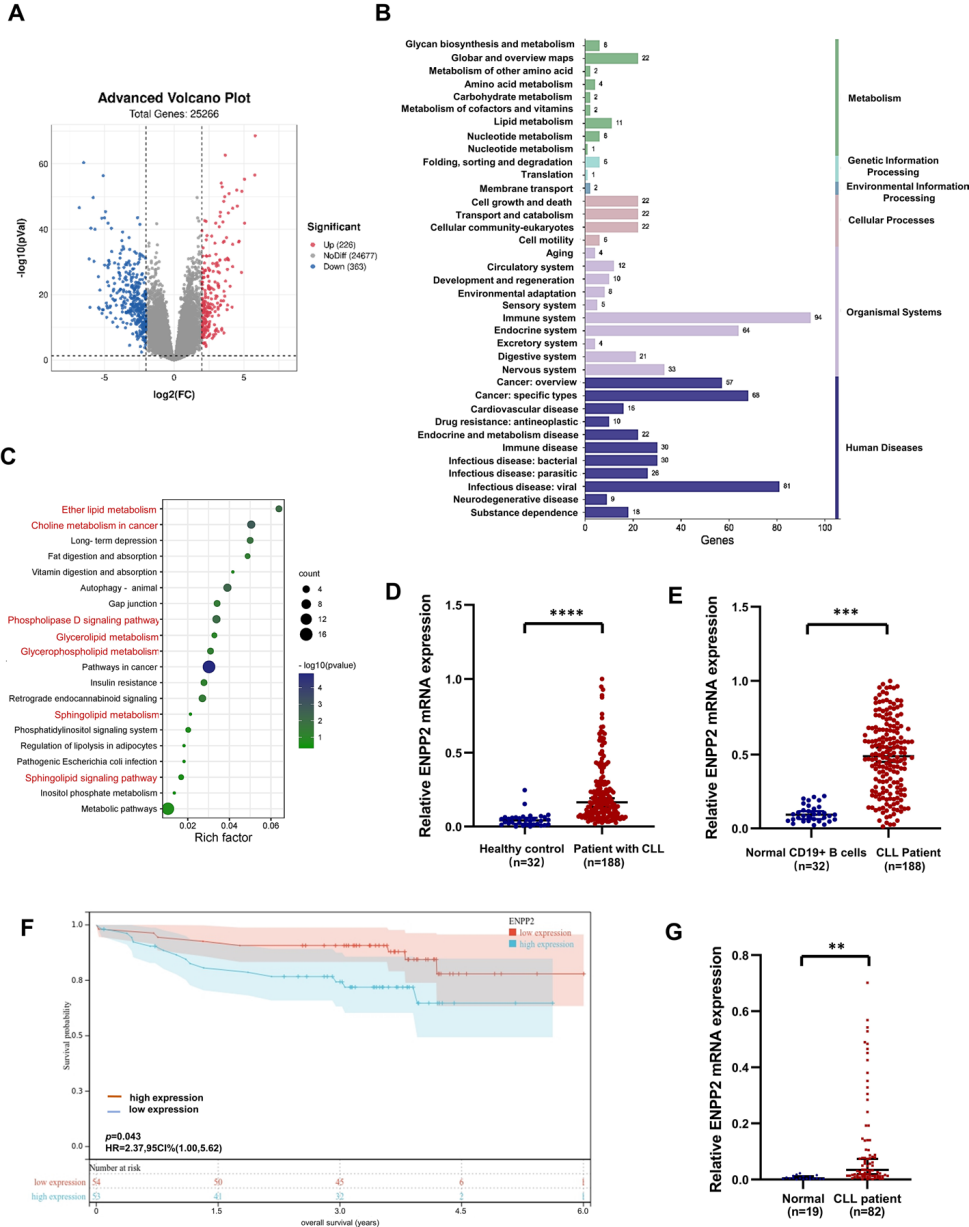
Combined transcriptome and metabolome data analysis revealed abnormal expression of ENPP2 in CLL. **A** Sequencing data GSE50006 was downloaded from the GEO database with the screening condition |log2(fold change) |> 2, *p* < 0.001. Significantly up-regulated genes are shown in red, significantly down-regulated genes are shown in blue, and non-significantly different genes are shown in gray. **B** Enrichment analysis of differential genes was performed. **C** KEGG enrichment was performed on 20 genes. **D**, **E** ENPP2 expression was significantly upregulated in the CLL public database. The analysis was based on GSE5006 and GSE31048, respectively.** F** Overall survival (OS) curves of patients with CLL based on ENPP2 stratified expression of GSE22762. **G** ENPP2 mRNA was elevated in CLL progenitor cells compared with normal CD19^+^ B cells. Data are shown as mean ± SD. *p < 0.05, **p < 0.01, ***p < 0.001, ****p < 0.0001

The genomic microarray profile GSE50006 was performed for transcriptome analysis. The DEGs screening threshold was set to |log2(fold change) |> 0.25, adjusted to *p* < 0.01. In this study, 539 DEGs were identified and 257 of the pathways were enriched, as depicted in Fig. 2A, B. In our current study, the pathway analysis based on metabolomics and transcriptomics data produced 20 KEGG pathways (Fig. S1D). As depicted in Fig. 2C, these pathways cover a variety of metabolic processes, including phosphatidylinositol signaling system, phospholipase D signaling pathway, sphingolipid signaling pathway, choline metabolism in cancer, pathways in cancer, inositol phosphate metabolism, glycerophospholipid metabolism, glycerolipid metabolism, ether lipid metabolism, sphingolipid metabolism, fat digestion and absorption, and regulation of lipolysis in adipocytes. The details are shown in Table 1.

**Table 1:** Integrating metabolomics and transcriptomics for KEGG pathway enrichment and relevant genes

Pathway	*p*-Value	Corrected *p*-value	Gene symbol
Gap junction	0.038993	0.074212	TUBB6|PDGFD|PRKACB
Autophagy—animal	0.00489	0.019897*	IGF1R|DDIT4|EIF2AK3|HIF1A|PRKACB
Phosphatidylinositol signaling system	0.200611	0.255915	PIK3C2B|DGKG
Phospholipase D signaling pathway	0.008727	0.028197*	CYTH3|RAPGEF3|PDGFD|DGKG|IGH
Sphingolipid signaling pathway	0.260831	0.315672	S1PR5|TNFRSF1A
Choline metabolism in cancer	0.001694	0.010158*	**CHPT1|PDGFD|DGKG|HIF1A|FOS**
Pathways in cancer	1.37E-05	0.000323**	CDKN2B|IGF1R|FOS|GNB4|PRKACB|RXRA|HIF1A|SMAD3|IL6|DLL1|JUP|ARAF|CDK6|IL15|LEF1|MYC
Insulin resistance	0.063329	0.104514	IL6|SOCS3|TNFRSF1A
Pathogenic *Escherichia coli* infection	0.369197	0.410993	TUBB6
Inositol phosphate metabolism	0.460569	0.485242	PIK3C2B
Metabolic pathways	0.210821	0.263247	PLD4|CD38|GCNT1|GPT2|AASS|NT5E|HACD1|PIK3C2B|MGAT3|RRM2|CHDH|CHPT1|LARGE1|DGKG|CSGALNACT1
Glycerophospholipid metabolism	0.04923	0.086061	PLD4|CHPT1|DGKG
Glycerolipid metabolism	0.094317	0.147409	MGAT3|DGKG
Ether lipid metabolism	0.008024	0.027446*	**PLD4|CHPT1|ENPP2**
Sphingolipid metabolism	0.363591	0.415781	SGPP2
Fat digestion and absorption	0.048439	0.085311	SCARB1|MGAT3
Vitamin digestion and absorption	0.185855	0.240998	SCARB1
Regulation of lipolysis in adipocytes	0.369197	0.410993	PRKACB
Retrograde endocannabinoid signaling	0.036793	0.071173	GNAO1|GABRB2|GNB4|PRKACB
Long-term depression	0.015044	**0.03859**	**GNAO1|IGF1R|ARAF**

The blod values means statistically significant. *p < 0.05, **p < 0.01

By performing further analysis of the metabolism-related pathways on the basis of the corrected *p*-value, the choline metabolism pathway and the ether ester metabolism pathway were screened out. According to the transcriptome study refer to the two paths corresponding to the gene differences, namely cholinephosphotransferase-1 (CHPT1), platelet-derived growth factor-D (PDGFD), diacylglycerol kinase gamma (DGKG), hypoxia-inducible factor-1A (HIF-1A), FOS proto-oncogene (FOS), phospholipase D (PLD4), and ectonucleotide pyrophosphatase/phosphodiesterase2 (ENPP2). We preliminarily analyzed the differential expression of the above genes between patients with CLL and normal controls through public databases, and found that there were no significant differential features except for ENPP2 (Fig. S1E), and after reviewing literature, we chose to carry out the next step of research on the mechanism of action of the differential gene ENPP2 in CLL.

### Increased expression of ENPP2 in CLL cells

In the gene databases GSE50006 and GSE31048, which include 376 patients with CLL, the data were normalized by extreme deviation, and the expression of ENPP2 was significantly higher than in normal group (Fig. 2D, E). On the basis of statistical data from GSE22762, the Kaplan–Meier method observed that exhibiting high levels of ENPP2 expression experienced a considerably diminished overall survival (Fig. 2F). Additionally, specimens from patients with CLL were selected for qRT-PCR analysis, which demonstrated that the expression level of ENPP2 in CLL specimens was significantly higher when compared with the normal group (Fig. 2G). Moreover, compared with B cells from healthy volunteers, ENPP2 messenger RNA (mRNA) expression in MEC-1 was significantly higher than that in B cells (Fig. S1F).

### RNA sequencing analysis for ENPP2 functional enrichment in CLL cells

**Fig. 3:**
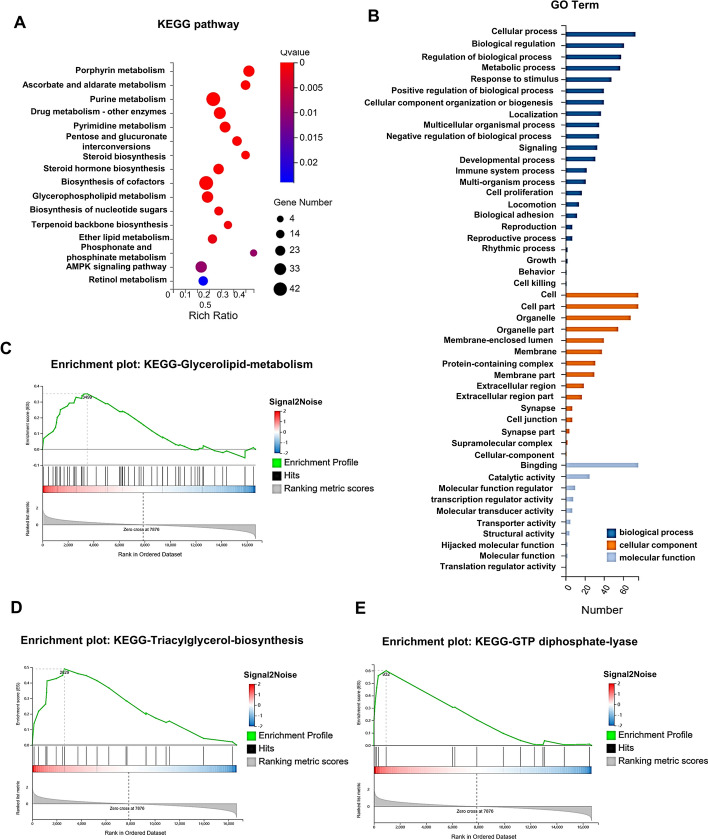
RNA-seq analysis of ENPP2 between ShControl and ShENPP2 cells. **A** KEGG enrichment analysis. **B** GO terms analysis of differently expressing genes. **C**–**E** GSEA analysis of differential gene expression correlated with ENPP2 was performed. NES, normalized enrichment score

To investigate the attributes of ENPP2, RNA-sequencing was performed on MEC-1 cells transfected with both ShControl and ShENPP2#2. The results of our study, depicted in Fig. 3A, indicate that ENPP2 was concentrated in pathways linked to metabolisms, such as the TCA cycle, ether lipid metabolism, and glycerophospholipid metabolism, through KEGG analysis. Gene ontology (GO) analysis revealed that ENPP2 is intimately involved in metabolic processes, cellular processes, and biological regulation (Fig. 3B). Gene set enrichment analysis (GSEA) revealed that ENPP2 was primary enriched in glycerolipid metabolism, triacylglycerol, and GTP diphosphate lyase (Fig. 3C–E). Taken together, ENPP2 may promote the occurrence of CLL by regulating lipid metabolic pathways.

### ENPP2 regulates the proliferation, apoptosis, and cell cycle of CLL cells

**Fig. 4:**
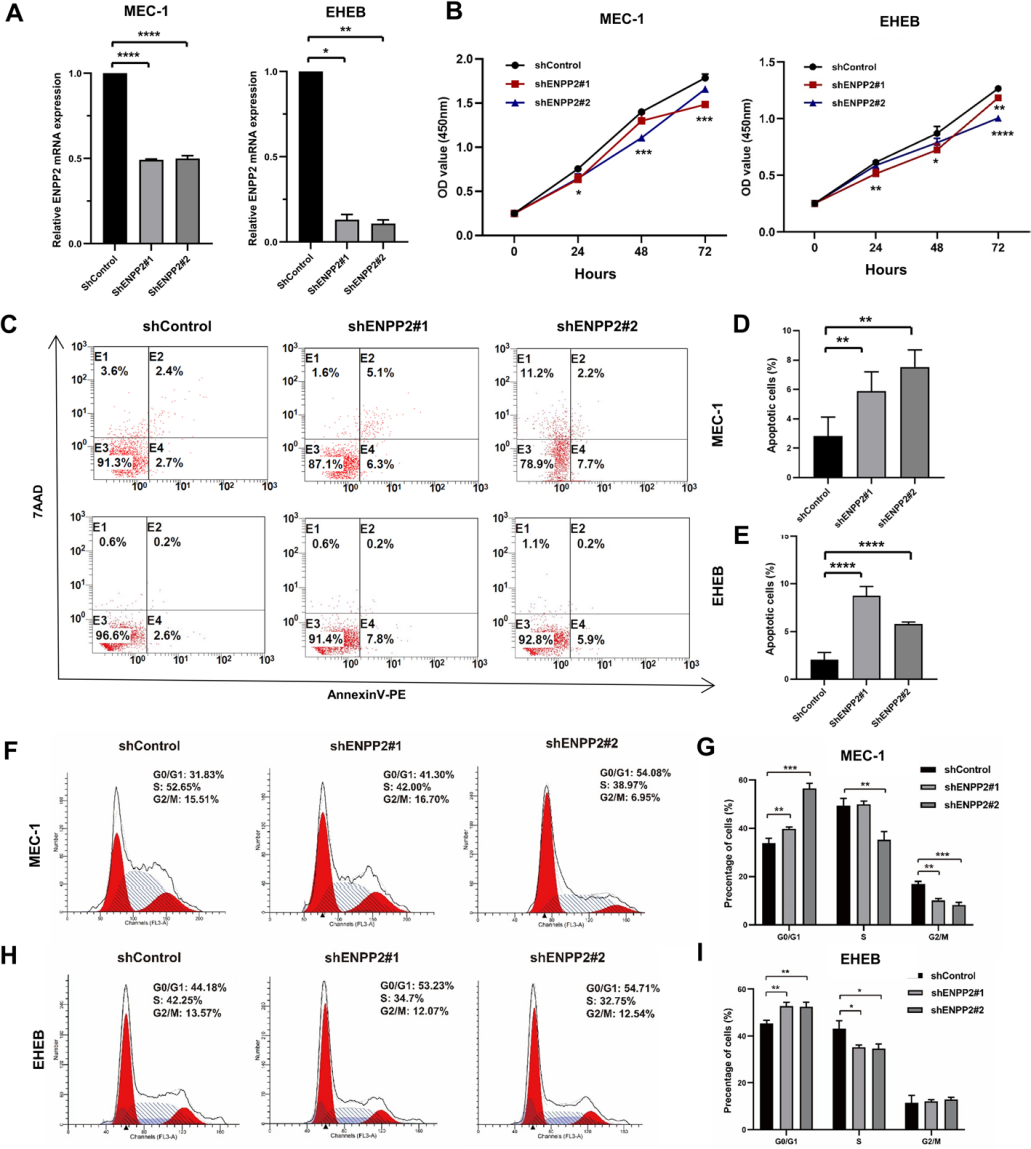
ENPP2 knockdown restrained the survival of CLL cell lines. **A** qRT-PCR assay for knockdown efficiency. **B** Proliferation curves of ENPP2 knockdown cells and control cells. **C**–**E** Flow cytometry detection of apoptosis after ENPP2 knockdown. **F**–**I** Flow cytometry detection of cycle distribution after ENPP2 knockdown and the relative proportions of cells in different cell cycle phases. Data are shown as mean ± SD. *p < 0.05, **p < 0.01, ***p < 0.001, ****p < 0.0001

To confirm the results of our bioinformatics analysis, we conducted functional experiments in CLL cells to investigate the role of ENPP2. ENPP2 was successfully silenced by ShENPP2#1 and ShENPP2#2 in MEC-1 and EHEB cells (Fig. 4A), and verified the decreased expression of ENPP2 protein (Fig. S3A). We determined that downregulation of ENPP2 indirectly inhibited CLL cell proliferation through CCK-8 assays (Fig. 4B). Furthermore, through Annexin V-PE/7AAD assay, we observed a notable increase in apoptosis of shENPP2 transfected cells (Fig. 4C–E). Additionally, we monitored the cell cycle of downregulated ENPP2 cells and found that they exhibited a significant G0/G1 phase block compared with control cells (Fig. 4F–I). The results highlight that the ENPP2 contributes significantly to the survival of CLL cells through its ability to inhibit apoptosis and facilitate the progression of cells from the G0/G1 phase. To further verify the biological function of ENPP2, we constructed overexpressed plasmids (Fig. S2A). In contrast, ENPP2 overexpression promoted cell proliferation, reduced the proportion of apoptotic cells and accelerated the cell cycle (Fig. S2B–D).

### Targeted inhibition of ENPP2 by PF-8380 exerted anti-tumor activity in CLL cells

**Fig. 5:**
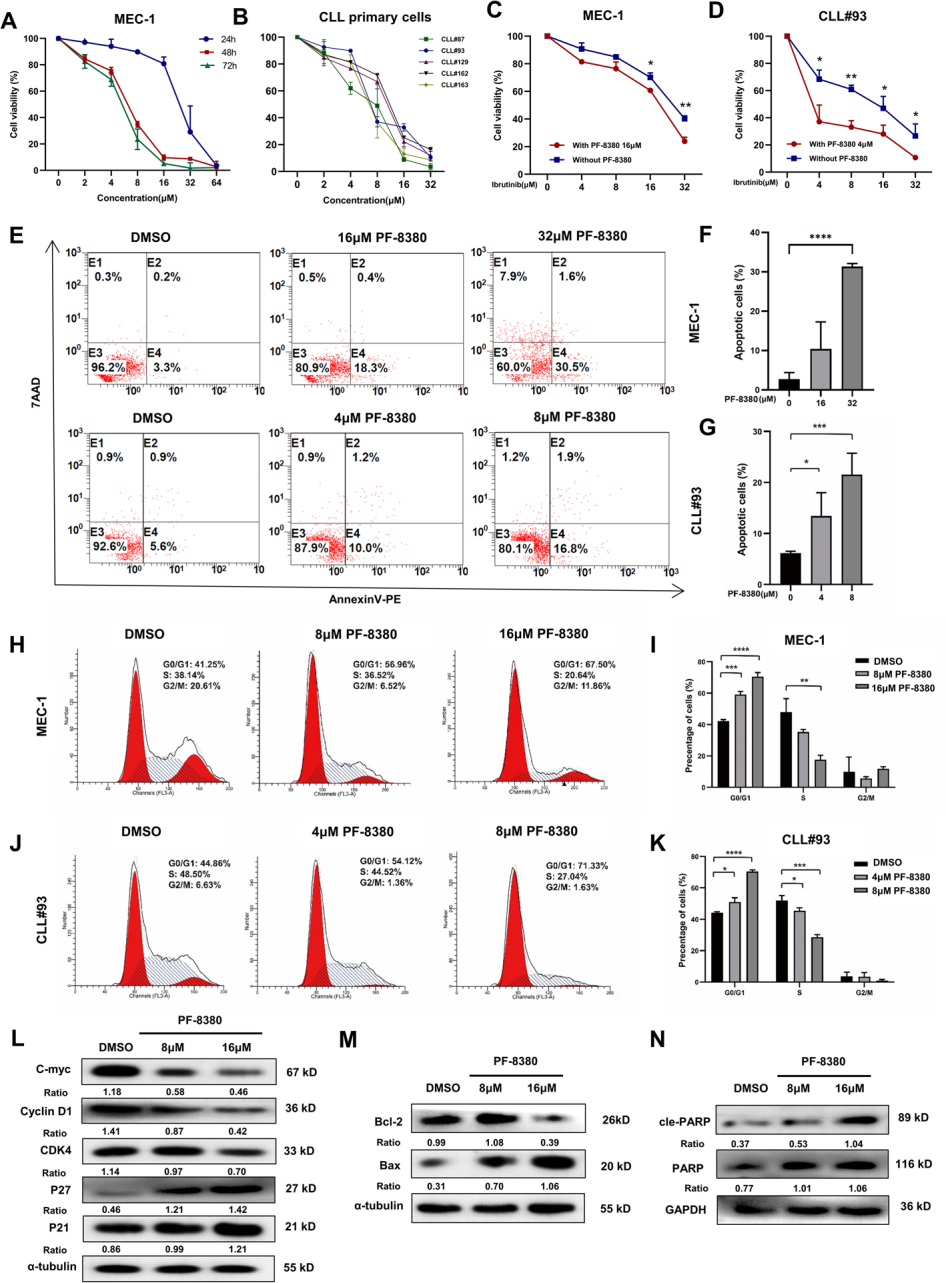
Effect of ENPP2 target inhibitor PF-8380 in CLL cells. **A** CCK8 assay to detect the survival rate of CLL cell line MEC-1 treated with different concentrations of PF-8380. **B** CCK8 assay to detect the survival rate of CLL primary cells treated with different concentrations of PF-8380. **C** CCK8 assay to detect the survival rate of MEC-1 cells in combination with ibrutinib. **D** CCK8 assay to detect the cell survival rate of CLL primary cells treated with ibrutinib. **E**–**G** Representative dot plots generated by flow cytometry analysis of PF-8380 groups versus negative control. **H**–**K** Representative results for the cell cycle distributions with PF-8380. **L** Detection of cycle-associated protein expression levels in MEC-1 cells. **M**, **N** Detection of apoptosis-associated protein expression levels in MEC-1 cells. Data are shown as mean ± SD of at least three independent experiments. *p < 0.05, **p < 0.01, ***p < 0.001

ENPP2 inhibitor PF-8380 decreased proliferation of MEC-1 cells in dose-dependent and time-dependent manner (Fig. 5A). PF-8380 also impeded cell viability of CLL primary cells at micromolar concentration (Fig. 5B). In addition, ibrutinib supplementation with 16 μM or 4 μM PF-8380 increased cytotoxicity to CLL cells (Fig. 5C, D). We used the combined index to determine the synergism of the two drugs. According to the judgment method of Soriano et al., 0.9 ≤ combined index ≤ 1.1 is superimposed, 0.8 ≤ combined index< 0.9 is low synergism, 0.6 ≤ combined index < 0.8 is moderate synergism, 0.4 ≤ combined index < 0.6 is high synergism, and 0.2 ≤ combined index < 0.4 is strong synergism. We calculated a combined index of 0.74 in the MEC1 cell line and 0.45 in CLL#93 for the combination of ibrutinib and PF8380, which is sufficient to show that the two drugs are synergistic not only in the CLL cell line but also in primary cells.

Moreover, the amount of apoptotic cells increased with the increase of PF-8380 concentration after 24 h flow cytometry analysis of MEC-1 and primary CLL cells treated with PF-8380 (Fig. 5E–G). Compared with DMSO treatment, PF-8380 also induced the increase of G0/G1 phase cells (Fig. 5H–K). Western blotting analysis showed that with the increase of PF-8380 concentration, the levels of cyclin-related proteins, including C-myc, Cyclin D1, CDK4, P21, and P27 (Fig. 5L) and apoptosis-related proteins, such as Bcl-2, Bax, and cle-PARP changed (Fig. 5M, N). Taken together, PF-8380 exerts therapeutic potential by inhibiting CLL cell survival and cell cycle, enhancing apoptosis and chemosensitivity.

### ENPP2 regulates lipid metabolism in CLL

**Fig. 6:**
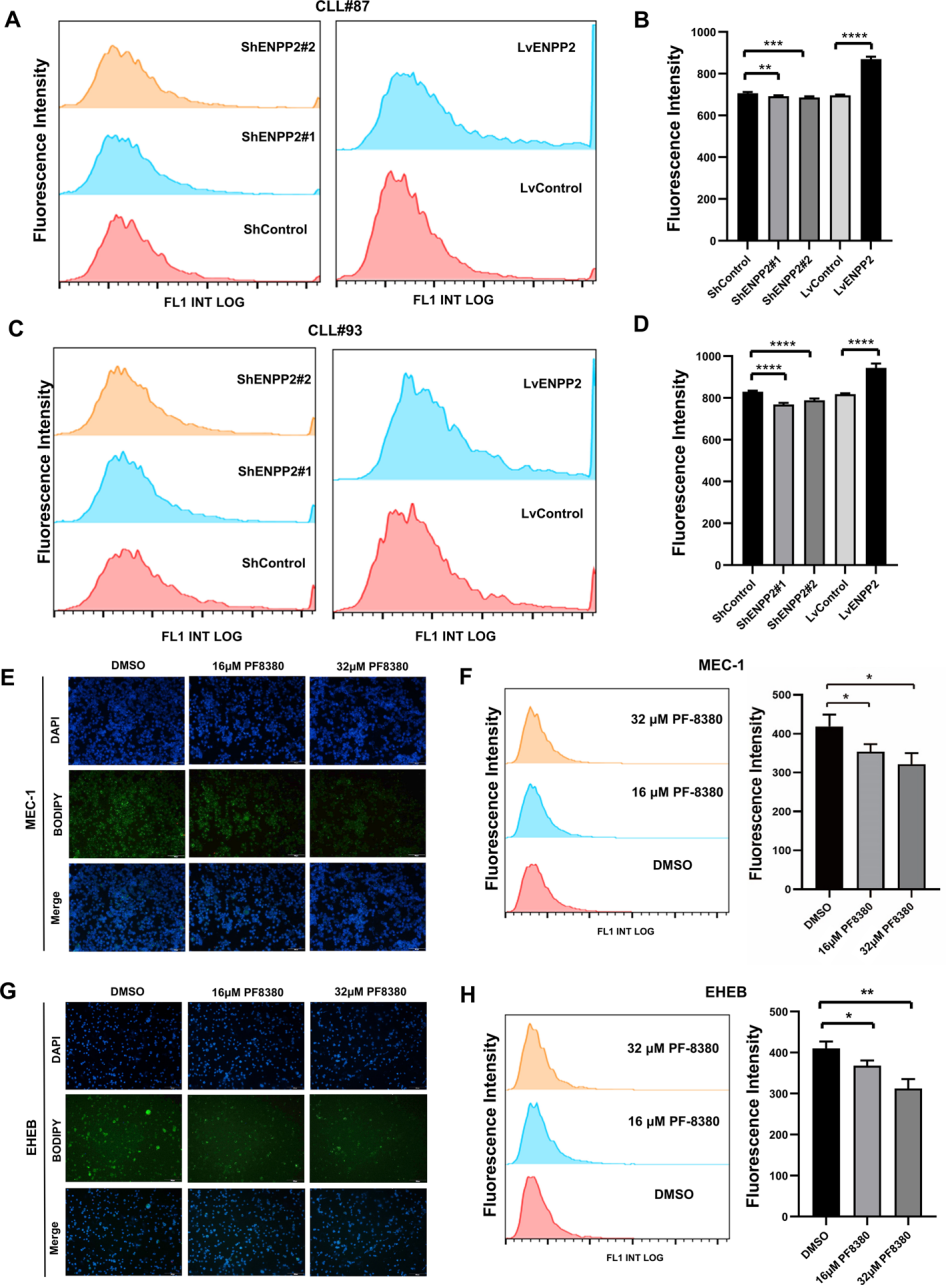
Effect of ENPP2 knockdown and overexpression on intracellular lipids. Alterations of lipids in cells treated with PF-8380. **A**–**D** Detection of primary patient cell lipid content by bodipy staining and flow cytometry. **E** Detection of intracellular lipid content of MEC-1 by Bodipy staining (lipids stained green and cell nuclei stained blue; scale bars, 100 µm). **F** Quantification of intracellular lipid content of MEC-1 by flow cytometry. **G** Detection of intracellular lipid content of EHEB by Bodipy staining (lipids stained green and cell nuclei stained blue; scale bars, 100 µm). **H** Quantification of intracellular lipid content of EHEB by flow cytometry. Data are shown as mean ± SD of at least three independent experiments. *p < 0.05, **p < 0.01, ***p < 0.001

Previous combined metabolomics and transcriptional analyses, as well as RNAseq, provide evidence that ENPP2 may act as a regulatory factor for lipid metabolism in CLL, and holds significance in the lipid metabolic process. To test this hypothesis, bodipy staining (Fig. S3C, D) showed increased lipid accumulation in ENPP2 overexpressed CLL cell lines and significantly reduced lipid accumulation in ENPP2 knockout cells. In addition, quantitative analysis of bodipy staining was performed by flow cytometry, and the results were consistent with the above (Fig. S4A–D). We additionally validated this finding in CLL primary patients (Fig. 6A–D).

### Effect of targeted inhibition PF-8380 on lipid metabolism in CLL cells

To explore the effect of ENPP2-targeted drug PF-8380 on the lipid metabolism of CLL, we treated CLL cell lines with 16 μM and 32 μM, respectively. We stained them with bodipy (Fig. 6E, G). The study findings suggest that as drug concentration increased, the intracellular lipid deposition decreased gradually. The quantitative treatment of bodipy staining by flow cytometry showed the same results as before (Fig. 6F, H). Additionally, we measured the content of TG in the cells treated with the drug. The observed decline in TG content within the cells was found to be directly proportional to the increase in drug concentration (Fig. S3B). This trend is congruent with the results obtained via Bodipy staining. Taken together, the ENPP2 targeted inhibitor PF-8380 could alter the disease course by regulating lipid metabolism.

### ENPP2 functions through the AMPK/SREBP1/FAS pathway

**Fig. 7:**
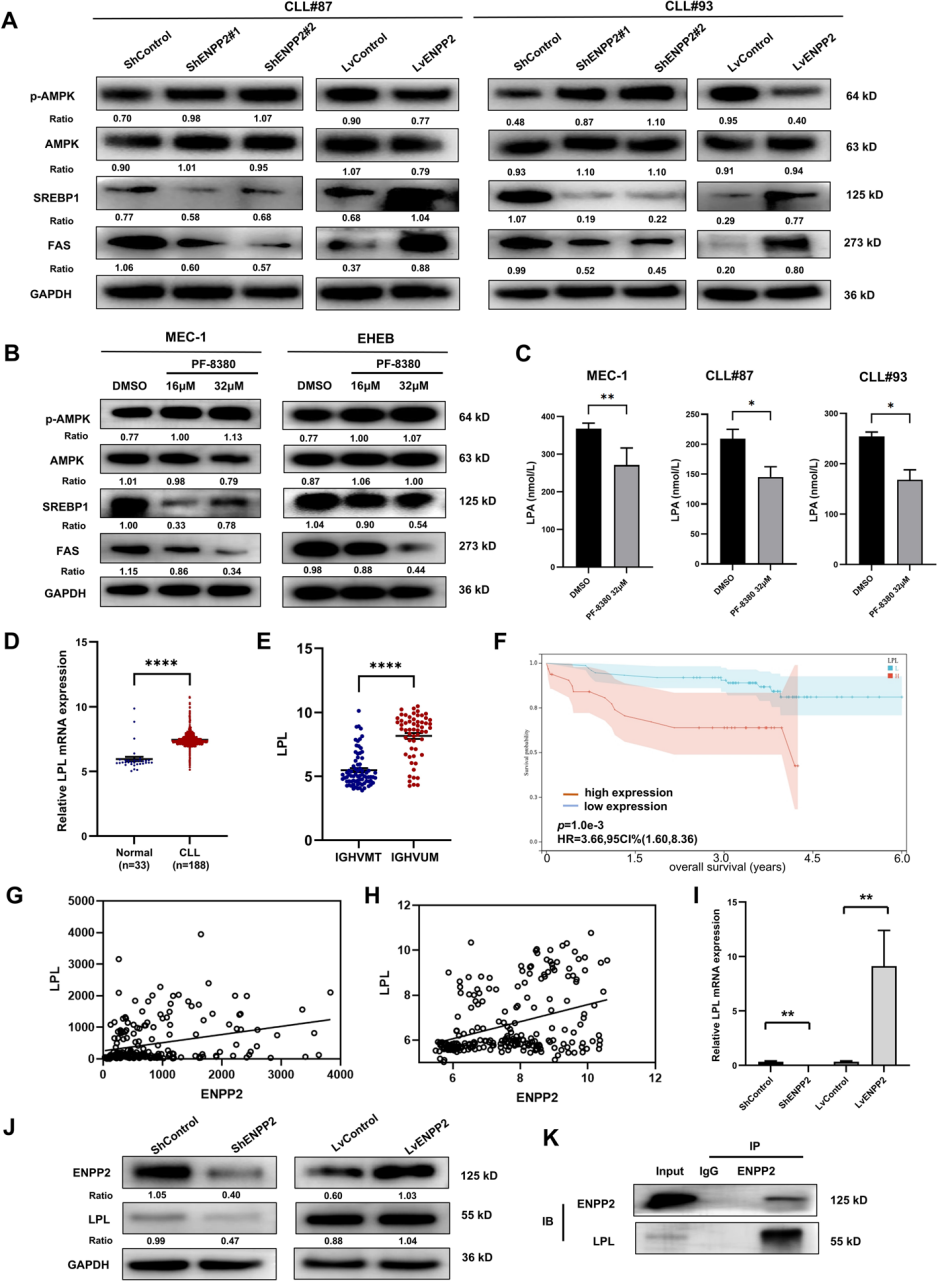
ENPP2 regulated AMPK signaling pathway and interacted with LPL. **A** Protein expression of p-AMPK, AMPK, SREBP1, and FAS in patient with primary CLL. **B** Protein expression of p-AMPK, AMPK, SREBP1, and FAS with PF-8380 treatment. **C** MEC-1, patients with CLL cells were treated with 32 μM PF8380 for 24 h and the cell supernatant LPA levels were measured by ELISA. **D** LPL was markedly upregulated in CLL public database. Analyses were on the basis of GSE31048. **E** Patients with CLL with unmutated IGHV presented high LPL expression (GSE69034). **F** Kaplan–Meier survival curves of patients with CLL from GSE22762 with stratified LPL expression. **G**, **H** Correlation between ENPP2 and LPL mRNA expression in patients with CLL from GSE50006 and GSE31048. **I** qRT-PCR was performed to detect LPL mRNA content in ENPP2 knockdown and overexpression cells. **J** Western Blot assays for the amount of LPL protein in ENPP2 knockdown and overexpression cells. **K** Co-immunoprecipitation demonstrated that ENPP2 and LPL could be co-precipitated. Data are shown as mean ± SD of at least three independent experiments, *n* = 3. *p < 0.05, **p < 0.01, ***p < 0.001

We then considered how ENPP2 regulates the process of lipogenesis in CLL. On the basis of RNA sequencing results, we became attracted to AMP-activated protein kinase (AMPK), which is a central player in metabolism[24] and negatively correlates with tumor progression and genesis[25, 26]. AMPK/SREBP1/FAS pathway is one of the key pathways for intracellular lipogenesis. AMPK regulates the expression of adipogenic genes through the sterol regulatory elements binding transcription factor 1(SREBP1) transcription factor. We examined the protein levels of p-AMPK, AMPK, SREBP1, and fatty acid synthase (FAS) to elucidate the molecular mechanism of ENPP2 involvement in cellular lipid metabolism. The results showed that the AMPK phosphorylated form was significantly increased in ENPP2 knockdown cells compared with control. Moreover, ENPP2 knockdown significantly decreased SREBP1 and FAS proteins. ENPP2 overexpression showed results corresponding to knockdown cells (Fig. S4E). We additionally validated this finding in patients with primary CLL (Fig. 7A). In addition, we treated MEC-1 with 16 μM and 32 μM PF-8380, which showed enhanced AMPK phosphorylation and attenuated SREBP1 and FAS (Fig. 7B). ENPP2 is a secreted lysophospholipase D that promotes the hydrolysis of extracellular lysophosphatidylcholine (LPC) to lysophosphatidic acid (LPA) [27]. Therefore, we assayed the LPA content in cell supernatants after drug treatment utilizing ELISA. The results upon calibration for cell counts showed that both in the CLL cell line and primary cells from different patients with CLL, LPA in the cell supernatant was significantly decreased after treatment with the targeted inhibitor PF-8380 compared with the DMSO control (Fig. 7C).

### ENPP2 interacted with LPL in CLL cells

**Fig. 8:**
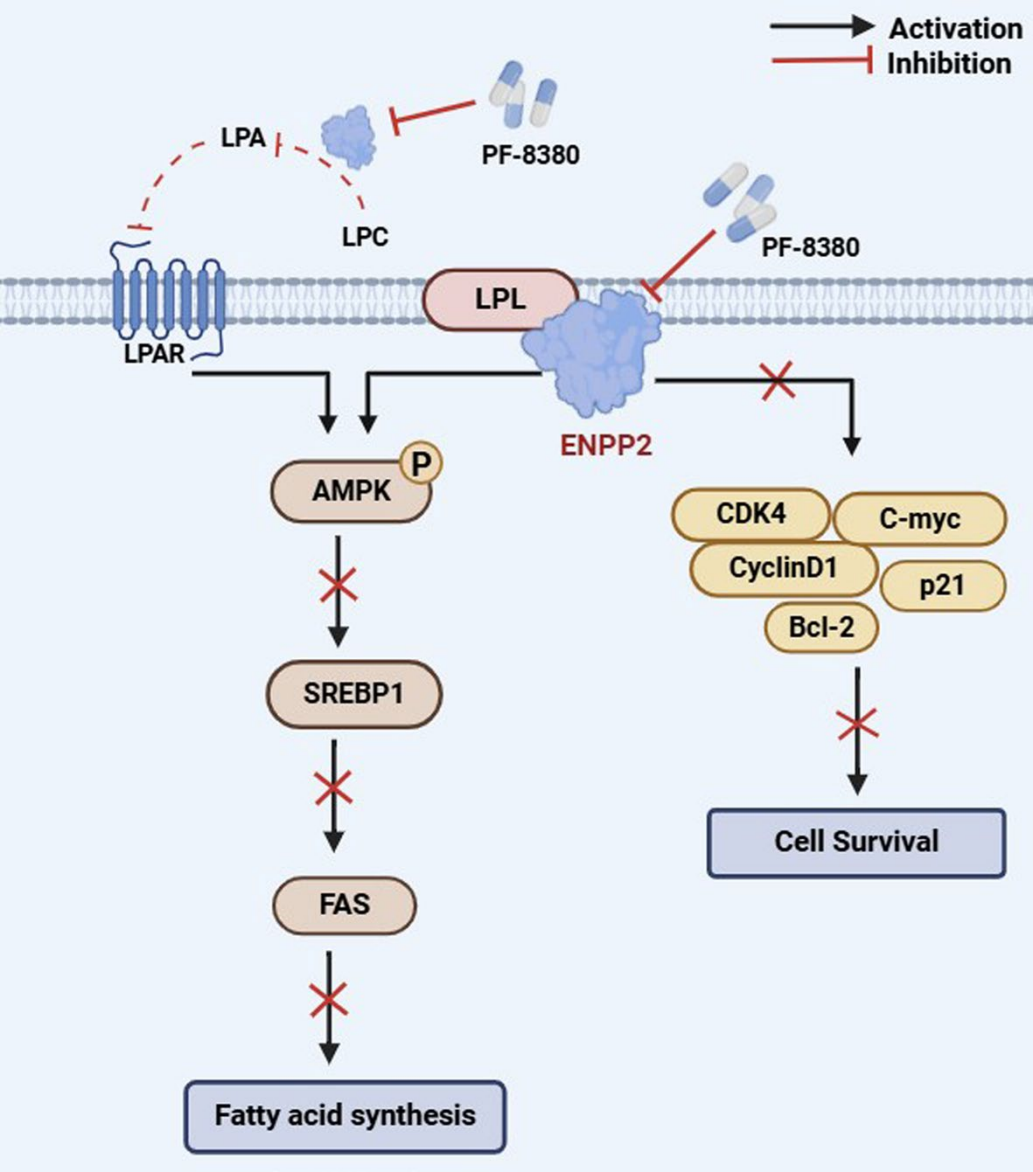
Schematic model of ENPP2 mediated lipid metabolism

Lipoprotein lipase (LPL) has been identified as a crucial driver in the metabolic processes of CLL cells by facilitating the absorption of lipoprotein [28–30]. The GSE31048, GSE69034, and GSE22762 normalized microarray RNA-seq data was download from the GEO database. We verified that LPL expression is increased in patients with CLL, and LPL expression was also significantly associated with unmutated IGHV genes (Fig. 7D, E). The results of Kaplan–Meier survival curve analysis revealed a significant correlation between high LPL expression and poor prognosis in patients with CLL (Fig. 7F). Meanwhile, in GEO database (GSE50006 and GSE31048), ENPP2 expression exhibited a significant positive correlation with LPL (Spearman: *r* = 0.3057, *p* < 0.0001; Spearman: *r* = 0.3653, *p* < 0.0001; Fig. 7G–H). It is hypothesized that ENPP2 may regulate CLL lipid metabolism through LPL. We transfected ShENPP2 and LvENPP2 into CLL cell lines to detect LPL levels. The results showed a significant positive correlation between LPL and ENPP2 expression, evident through analysis of mRNA and protein levels (Fig. 7I–J). Subsequently, further Co-IP experiments revealed potential interactions between ENPP2 and LPL in CLL cells (Fig. 7K). Our results provide evidence that ENPP2 modulates LPL expression in CLL. Taken together, the catalytic function of ENPP2 in CLL tumorigenesis was preliminarily elucidated (Fig. 8).

## Discussion

In this study, lipidomic analysis suggested differences in lipid metabolites between patients with CLL and age-matched healthy controls. The correlation between lipid metabolism and CLL has been substantiated. Considering that the age of onset of patients with chronic lymphocytic leukemia is commonly high, and lipid metabolism may change with increasing chronological age. Therefore, we performed a correlation analysis between patients’ age and common lipid metabolism indexes before analysis to exclude the interference of age. We used an untargeted quantitative metabolomics approach to examine and contrast the distinct serum metabolic profiles of patients with CLL and healthy individuals. We then validated the selected metabolites and corresponding pathways by transcriptomic data, thus identifying altered biological processes or metabolic features in patients with CLL. A total of 52 differential metabolites and 539 differential genes were defined, and three metabolites (PC O-24:2_18:2, PC O-35:3, LPC 34:3) were selected as biomarkers for CLL diagnosis on the basis of the ROC curve area.

Additionally, further transcriptomic associations suggested that the CLL DEGs were significantly enriched in lipid metabolism pathways. On the basis of our analysis of CLL metabolomics combined with bioinformatics, we propose that ENPP2 might be closely related to the production of lipid metabolites and have important functions in lipid metabolism in CLL [31–36]. ENPP2 has been documented to promote coronary atherosclerosis by mediating LDL production through the generation of LPA 20:4, 16:0, and 18:1 and by inducing CXCL1 expression [31]. Our present study represents the role of ENPP2 in the pathogenesis of CLL, which is significantly expressed in patients with CLL, and predicted poorer survival and prognosis. Further, it was demonstrated that ENPP2 is involved in lipid metabolic pathways in CLL and promote cell survival by AMPK pathway to promote lipid deposition.

Cancer cells require metabolic reorganization to improve their value-added and survival rates compared with normally differentiated cells. Previous studies have found that lipid metabolism is important in tumorigenesis, progression and metastasis. Disturbances in lipid metabolism may induce abnormal gene expression and lead to abnormal signaling pathways [37]. Altered lipid metabolism is closely related to pathogenic processes and could give rise to distinct disease biomarkers with diagnostic, prognostic, and predictive capabilities. Untargeted metabolomics has emerged as an essential avenue for exploring lipid metabolism profiles [38–42]. The investigation of the metabolic changes in CLL cells has revealed their ability to store lipids and derive chemical energy from free fatty acids, similar to adipocytes [43]. Lipid droplet vesicles are present within the cytoplasm of CLL B cells, and upon incubation with free fatty acids, an upsurge in their metabolic rate could be witnessed [29]. Pallasch’s colleagues have identified significantly elevated levels of lipase-related genes and triglyceride-specific lipase activity in CLL B cells compared with normal CD5^+^ B cells. Notably, the inhibition of lipase activity has been shown to increase CLL cell apoptosis [44].

ENPP2 as an adipose-derived secretory enzyme, controls adipose expansion, a fat brown supply and energy expenditure [11]. In recent years, it has been shown that ENPP2 is closely correlated with obesity and disorders of glucolipid metabolism in obese individuals [45]. It is considered a possible target for the treatment of obesity-related diseases. Adipocyte ENPP2 expression was accompanied by a substantial increase in adipogenesis in individuals exhibiting type II diabetes associated with obesity [46]. Prior research has demonstrated the potential of ENPP2 as a prognostic biomarker in various cancers [47], including breast and liver cancer [48, 49]. Cholia and his colleagues found that ENPP2 enhances the aggressive potential of glioblastoma [50]. Through a comprehensive analysis inclusive of RNA sequencing, this study sheds light on the regulatory role of ENPP2 in CLL. Furthermore, this investigation identified ENPP2 as an important biomarker of prognosis in CLL. Our analysis has revealed a dysregulated expression of ENPP2 in CLL, and a strong correlation between elevated ENPP2 expression and patient survival, as demonstrated in GSE22762, suggesting a potential role for ENPP2 in CLL progression. Further validation studies are required to confirm its predictive significance. Our findings indicate that the silencing of ENPP2 results in decreased cell proliferation, enhanced apoptosis, and G0/G1 cell cycle arrest.

To elucidate the molecular mechanisms involved in lipid metabolism by ENPP2, we examined the degree of AMPK protein phosphorylation and downstream target gene regulation. AMPK is engaged in energy sensing and homeostasis regulation in vivo, and performs a crucial function in lipid regulation [51]. AMPK is believed to be fundamental for lipid metabolism through the regulation of fatty acid synthesis and regulation [52, 53]. Prior research has demonstrated that AMPK could modulate SREBP1 and FAS, thereby impacting adipogenesis and lipid metabolism [24]. ENPP2 is more commonly reported for the formation and cellular function of its product LPA, which activates multiple signaling pathways, such as MEK/ERK, NF-kB, and CREB pathways, via G protein-coupled receptors [36]. Coincidentally, LPA has been reported to stimulate glucose uptake and regulate AMPK phosphorylation. This connection may provide a novel insight into the regulation of lipid metabolism by ENPP2. Our research has demonstrated that the reduction of ENPP2 inhibits lipid accumulation by augmenting AMPK phosphorylation and reducing the level of SREBP1 and FAS. ENPP2 regulation of the AMPK/SREBP1/FAS signaling pathway may be an effective mechanism for anti-lipogenic effects in CLL cells.

LPL is an enzyme normally expressed in adipocytes and muscle cells and is essential for the metabolism of free fatty acids [54]. It has been demonstrated that it is not expressed in normal lymphocytes, but its expression is increased in CLL cells. It has also been meaningfully associated with the prognosis of CLL, and high expression levels of LPL are usually associated with poorer clinical outcomes [55, 56]. LPL induces lipoprotein storage in CLL cells and reprograms CLL cells to preferentially use lipids as an energy source. It seems to result in a higher cell survival rate [44, 54]. Metabolic reprogramming is initiated as CLL cells increase their demand for energy and metabolites to meet their rapid proliferation and survival [2]. ENPP2 expression was increased in CLL cells. Consistent with our hypothesis, it has been observed that the downregulation of ENPP2 demonstrates notable anti-leukemic properties and reduced the role of key kinases in the lipid metabolism pathway. In CLL cells, LPL expression was reduced after ENPP2 silencing, whereas LPL expression was enhanced after ENPP2 overexpression, suggesting a positive effect of ENPP2 on LPL expression. We hypothesized that ENPP2 might participate in cellular lipid metabolism by binding to LPL, thus regulating CLL cell growth. Therefore, we elucidated the interaction between ENPP2 and LPL through Co-IP experiments. Our results demonstrate that the aberrant lipid metabolism pathway involved in ENPP2 is involved in the regulation of CLL onset and development.

PF-8380 serves as a targeted inhibitor of ENPP2 and has been implicated in the pathogenesis and management of numerous diseases. Specifically, PF-8380 has been shown to elicit a reduction in tumor vascularity, delay tumor growth, and heighten radiosensitivity in glioblastoma [57]. In a mouse model of hepatic encephalopathy, PF-8380 has demonstrated the ability to mitigate neuroinflammation and enhance neurological function [58]. Studies undertaken by D’Souza and colleagues have demonstrated that 24-h incubation of adipocytes with PF-8380 resulted in increased production of peroxisome proliferator-activated receptor γ and downstream targets consequent to ENPP2 inhibition [59]. Nevertheless, the role of PF-8380 in the treatment of CLL warrants further exploration. We have demonstrated the antitumor effect of PF-8380 in CLL through in vitro experimentation, which confers a novel avenue for the treatment of this malignancy.

Over the past few years, targeted drug therapies have demonstrated remarkable therapeutic effects in CLL [60]. Although ibrutinib, a Bruton’s tyrosine kinase inhibitor, has displayed impressive efficacy in CLL treatment [61], its clinical resistance is still a significant challenge. Drug resistance and toxicity lead to poor clinical outcomes [62–64], which could be mitigated through the implementation of combination therapy aimed at reducing the incidence of drug resistance [65]. In our study, we observed that the ENPP2 targeted inhibitor PF-8380 exhibited positive antidrug resistance in CLL-targeted drug sensitivities, such as Ibrutinib, thus providing new prospects for clinical chemotherapy resistance. However, it is imperative to further investigate the mechanism of resistance and the clinical implementation of PF-8380 in the treatment of CLL.

## Conclusions

In summary, our investigation has screened differential metabolites of CLL and established a diagnostic model utilizing lipidomic. Furthermore, our results have highlighted the potential of inhibiting ENPP2 to impede the progression of CLL. Specifically, we have observed antitumor effects of PF-8380 in CLL, such as hindering cell survival, enhancing cell apoptosis, and blocking the cell cycle. Taken together, our findings suggest that ENPP2 serves as a promising target for targeted therapeutic interventions, potentially paving the way for an innovative approach to treating CLL.

## Supplementary Information

Additional file 1.

Additional file 2. Supplementary Fig S1. **A** LC-MS based PCA score plot. **B** PLS-DA score plot (R2 = 0.935, Q2 = 0.679). **C** PLS-DA model alignment test. Two coordinate points on the scoring plot are relatively far apart, indicating a significant difference between the two samples, and vice versa. The oval area represents the 95% confidence interval. **D** Higher expression of ENPP2 mRNA in CLL cell line MEC-1 than in normal CD19^+^ B cells was detected by qRT-PCR. **E** Venn diagram showing metabolomics and transcriptomics with 20 intersecting genes. **F** All integrated DEGs were analyzed for protein-protein interaction networks using the STRING database.

Additional file 3. Supplementary Fig. S2. **A** qRT-PCR detection of ENPP2 overexpression efficiency. **B** Proliferation curves of ENPP2 overexpression and control cells. **C**–**E** Flow cytometry detection of apoptosis after ENPP2 overexpression. **F** Flow cytometry detection of cycle distribution after ENPP2 overexpression and the relative proportions of cells in different cell cycle phases. Data were shown as the mean ± SD of at least three independent experiments. *p < 0.05, **p < 0.01, ***p < 0.001.

Additional file 4. Supplementary Fig. S3. **A** Protein expression following knockdown of ENPP2. **B** Detection of intracellular triglyceride content. **C**, **D** Quantification of MEC-1 and EHEB intracellular lipid content by bodipy staining (lipids stained green and cell nuclei stained blue; scale bars, 100 µm). Data were shown as the mean ± SD of at least three independent experiments. *p < 0.05, **p < 0.01, ***p < 0.001.

Additional file 5. Supplementary Fig. S4.** A**–**D** Detection of MEC-1 and EHEB lipid content by bodipy staining and flow cytometry (lipids stained green and cell nuclei stained blue; scale bars, 100 µm). **E** Protein expression of ENPP2, p-AMPK, AMPK, SREBP1, FAS in CLL cells.

Additional file 6. Supplementary Fig. S5. **A** The quantitative data of cycle-associated protein expression levels in MEC-1 cells. **B** The quantitative data of apoptosis-associated protein expression levels in MEC-1 cells. **C** The quantitative data of p-AMPK, AMPK, SREBP1, FAS in primary CLL patient. **D** The quantitative data of p-AMPK, AMPK, SREBP1, FAS with PF-8380 treatment. Data were shown as the mean ± SD of at least three independent experiments, n = 3. *p < 0.05, **p < 0.01, ***p < 0.001.

Additional file 7. Supplementary Table S1. Clinical information for CLL patients.

## Data Availability

The datasets used and/or analyzed in our study are available from the GEO database (https://www.ncbi.nlm.nih.gov/geo/) and the TCGA database (https://portal.gdc.cancer.gov/). The original images of western blotting analysis in this study are included in Additional file. The additional data supporting the findings of this study could be obtained from the corresponding author upon reasonable request.

## Abbreviations

CLL: Chronic lymphocytic leukemia
ENPP2: Ectonucleotide pyrophosphatase/phosphodiesterase 2
LPL: Lipoprotein lipase
AMPK: Adenosine 5′-monophosphate (AMP)-activated protein kinase
SREBP1: Sterol regulatory element-binding protein 1
FAS: Fatty acid synthase
IWCLL: International workshop on chronic lymphocytic leukemia
PBMCs: Peripheral blood mononuclear cells
OS: Overall survival
RNA-seq: RNA sequencing
GEO: Gene Expression Omnibus
KEGG: Kyoto Encyclopedia of Genes and Genomes
GO: Gene Ontology
qRT-PCR: Quantitative real-time polymerase chain reaction
MOI: Multiplicity of infection
CCK8: Cell Counting Kit-8
Co-IP: Co-Immunoprecipitation
