# Supplementary material for: ENPP2 promotes progression and lipid accumulation via AMPK/SREBP1/FAS pathway in chronic lymphocytic leukemia
Source: Cell Mol Biol Lett. 2024 Dec 27;29:159. doi: 10.1186/s11658-024-00675-6 (PMC11681649; doi:10.1186/s11658-024-00675-6)
Supplement: Supplementary file 1 — Additional file 1. [file 11658_2024_675_MOESM1_ESM.docx]

**supplementary materials**

Additional file 1: Materials and methods

**Univariate and multivariate analyses**

The data were normalized using the probabilistic quotient normalization (PQN) algorithm for all samples. Then quality control-robust spline batch correction (QC-RSC) was performed using QC samples. Metabolites with relative standard deviations (RSD) >30% in QC samples were removed. Univariate analysis was performed for metabolites, and statistical analysis was conducted utilizing the t-test. The results were analyzed as multiple tests, mainly using Benjamini-Hochberg (BH) correction to q-values. Multivariate analysis was performed for metabolites, mainly using partial least squares discriminant analysis (PLS-DA) to obtain each metabolite’s variable importance in projection (VIP). Volcano plot was mapped based on all detected ions and the distribution of these ions was shown. ggplot2, an R package, was implemented for visualization.

**Cell proliferation assays**

Experiments were performed using the Cell Counting Kit-8 (CCK-8) from Dojindo, Kumamoto, Japan. Following seeding of 10^4^ cells into 96-well plates, cells were incubated for 24, 48, and 72 h after corresponding treatments. Then, at predetermined time points, 10 μl of CCK-8 solution was added to each well and allowed to incubate for 1-4 h. Measurement of absorbance was conducted utilizing the SpectraMax M2 Microplate Reader from the company Molecular Devices located in CA, USA at a wavelength of 450 nm.

**Analysis of cell apoptosis and cell cycle**

The Annexin V-PE/7AAD Kit (BD Biosciences, Bedford, MA, USA) was utilized to examine cellular apoptosis. The Annexin V-PE/7AAD mix was added to the cell suspension and incubated for 15 minutes. Cells intended for cell cycle analysis were retrieved using PBS and subsequently immersed in 70% ethanol prior to being stored at a temperature of -20 °C overnight. The cells were exposed with PI/RNase Staining Buffer (BD Biosciences, Bedford, MA, USA) for 15 minutes. Detection was performed utilizing Navios flow cytometer from Beckman Coulter Inc. Data was analyzed using ModFit LT software.

**RNA isolation and quantitative real-time PCR**

Total RNA was purified by using RNAiso Plus (TaKaRa, Dalian, China). RNA concentration was measured via NanoDrop 2000 spectrophotometer (Thermo Fisher Scientific, WALTHAM, MA). Reverse transcription was taken by highvolume complementary DNA reverse transcription kit (TaKaRa, Dalian, China). Quantitative real-time polymerase chain reaction (qRT-PCR) was conducted through the Power SYBR™ Green PCR Master Mix (TaKaRa, Dalian, China) on a 7400 real-time PCR system based on the manufacturer’s instructions. Finally, the fold change representing the mRNA expression levels of genes was calculated in terms of 2− ΔΔCT. GAPDH served as an internal reference.

**Western blotting**

The cells protein was isolated by dissolving buffer solution with protease inhibitor (Beyotime, Shanghai, China) and placed on ice for 0.5 h. The bicinchoninic acid assay (BCA) method was put to measure protein concentration after centrifugation (Shenergy Biocolor, Shanghai, China). Proteins in equal amounts were added into the electrophoresis on sodium dodecyl sulfate-polyacrylamide gel hole and separated at 200 V for 30min. Then transfer the protein onto polyvinylidene fluoride membranes at 10 V for 30min. Next seal the membranes in 5% fat-free milk at room temperature for 1 h, and incubated overnight with primary antibodies of ENPP2(sc-374222, Santa Cruz Biotechnology), LPL (sc-373759, Santa Cruz Biotechnology), c-myc. Cyclin D1, CDK4, p21, p27, Bcl-2, Bax, PARP, cle-PARP (Cell Signaling Technologies, Beverly, MA, USA), α-actin, and GAPDH (Zhongshan Goldenbridge, Beijing, China) at 4 °C. After washing three times in Tris-buffered saline containing Tween (TBST), the membranes were incubated at room temperature for 1 h with appropriate secondary antibody (Zhongshan Goldenbridge, Beijing, China). After another three times washing, the chemiluminescence reagent (Pierce) was covered in the membranes for visualization at Bio-Rad Image Lab ™ (Bio-Rad, Hercules, CA) and the protein gray density quantification was analyzed via the ImageJ software. β-actin and GAPDH served as an internal reference.
