# Supplementary material for: ENPP2 promotes progression and lipid accumulation via AMPK/SREBP1/FAS pathway in chronic lymphocytic leukemia
Source: Cell Mol Biol Lett. 2024 Dec 27;29:159. doi: 10.1186/s11658-024-00675-6 (PMC11681649; doi:10.1186/s11658-024-00675-6)
Supplement: Supplementary file 7 — Additional file 7. Supplementary Table S1. Clinical information for CLL patients. [file 11658_2024_675_MOESM7_ESM.docx]

**Supplementary table 1.** **Chronic lymphocytic leukemia patients’ information.**

| Patient | Age | β2-MG | | LDH | TG | | TC | HDL | LDL | sdLDL | ApoA | ApoB | ApoA/ApoB | FFA | Lp(a) |
| --- | --- | --- | --- | --- | --- | --- | --- | --- | --- | --- | --- | --- | --- | --- | --- |
| CLL1 | 42 | 11.00 | 135.39 | | | 1.15 | 1.61 | 0.22 | 1.15 | 0.37 | 0.07 | 0.62 | 0.11 | 0.52 | 0.064 |
| CLL2 | 43 | 2.08 | 115.00 | | 0.97 | | 3.70 | 1.23 | 2.38 | / | 1.20 | 0.78 | 1.54 | / | 0.035 |
| CLL3 | 58 | 1.23 | 163.30 | | 0.75 | | 5.79 | 1.38 | 3.93 | 1.26 | 1.16 | 1.15 | 1.01 | 0.48 | 0.115 |
| CLL4 | 44 | 1.96 | 182.00 | | 0.58 | | 6.03 | 1.54 | 4.08 | 1.14 | 1.18 | 1.41 | 0.84 | 0.41 | 0.073 |
| CLL5 | 58 | 1.57 | 372.50 | | 1.91 | | 6.11 | 1.32 | 3.98 | / | 1.42 | 1.21 | 1.17 | 0.73 | 0.152 |
| CLL6 | 75 | 2.08 | 192.00 | | 1.29 | | 2.95 | 0.89 | 1.89 | 0.71 | 0.95 | 0.62 | 1.53 | 0.26 | 0.472 |
| CLL7 | 61 | 3.34 | 179.40 | | 0.67 | | 4.92 | 1.23 | 2.91 | 0.78 | 1.14 | 0.97 | 1.18 | 0.47 | 0.047 |
| CLL8 | 62 | 14.91 | 190.30 | | 1.06 | | 3.66 | 0.96 | 2.45 | 0.89 | 0.92 | 0.96 | 0.96 | 0.62 | 0.063 |
| CLL9 | 67 | 5.39 | 540.90 | | 1.59 | | 4.25 | 0.77 | 2.91 | 0.88 | 0.82 | 0.98 | 0.84 | 0.79 | 0.166 |
| CLL10 | 64 | 3.62 | 200.70 | | 1.37 | | 3.75 | 0.98 | 2.39 | 0.80 | 0.90 | 0.78 | 1.15 | 0.55 | 0.117 |
| CLL11 | 66 | 5.06 | 155.60 | | 0.70 | | 4.64 | 1.38 | 2.84 | 1.04 | 0.98 | 0.81 | 1.21 | 0.55 | 0.388 |
| CLL12 | 67 | 5.74 | 332.00 | | 1.31 | | 2.69 | 0.65 | 1.96 | 0.52 | 0.62 | 0.80 | 0.77 | 0.29 | 0.193 |
| CLL13 | 55 | 2.18 | 208.00 | | 1.09 | | 5.72 | 1.55 | 3.67 | 1.29 | 1.18 | 1.07 | 1.10 | 1.10 | 0.187 |
| CLL14 | 76 | 4.03 | 250.50 | | 0.75 | | 5.03 | 1.00 | 3.15 | 1.10 | 1.04 | 1.10 | 0.95 | 0.31 | 0.736 |
| CLL15 | 60 | 3.14 | 144.50 | | 0.79 | | 3.21 | 1.17 | 1.92 | 0.80 | 1.00 | 0.56 | 1.79 | 0.33 | 0.059 |
| CLL16 | 52 | 8.28 | 215.50 | | 1.22 | | 4.27 | 0.75 | 2.88 | 1.11 | 0.70 | 1.04 | 0.67 | 0.5 | 0.356 |
| CLL17 | 59 | 2.29 | 145.90 | | 1.66 | | 5.40 | 1.07 | 3.66 | 1.41 | 0.91 | 1.07 | 0.85 | 0.41 | 0.335 |
| CLL18 | 59 | 2.12 | 131.00 | | 0.87 | | 5.00 | 1.28 | 3.48 | / | 1.12 | 0.88 | 1.27 | / | 0.076 |
